# Ketoconazole induces reversible antifungal drug tolerance mediated by trisomy of chromosome R in *Candida albicans*

**DOI:** 10.3389/fmicb.2024.1450557

**Published:** 2024-07-30

**Authors:** Lijun Zheng, Yi Xu, Chen Wang, Liangsheng Guo

**Affiliations:** ^1^Department of Ultrasound Medicine, The Second Affiliated Hospital of Soochow University, Suzhou, China; ^2^Department of Pharmacy, The 960th Hospital of PLA, Jinan, China; ^3^Department of Obstetrics and Gynecology, The Second Affiliated Hospital of Soochow University, Suzhou, China

**Keywords:** *Candida albicans*, ketoconazole, antifungal agent, drug tolerance, chromosomal aneuploidy, Hsp90, calcineurin

## Abstract

**Background:**

The emergence of tolerance to antifungal agents in *Candida albicans* complicates the treatment of fungal infections. Understanding the mechanisms underlying this tolerance is crucial for developing effective therapeutic strategies.

**Objective:**

This study aims to elucidate the genetic and molecular basis of ketoconazole tolerance in *C. albicans*, focusing on the roles of chromosomal aneuploidy, Hsp90, and calcineurin.

**Methods:**

The wild-type *C. albicans* strain SC5314 was exposed to increasing concentrations of ketoconazole (0.015–32 μg/mL) to select for tolerant adaptors. Disk diffusion and spot assays were used to assess tolerance. Whole-genome sequencing identified chromosomal changes in the adaptors. The roles of Hsp90 and calcineurin in maintaining and developing ketoconazole tolerance were investigated using specific inhibitors and knockout strains.

**Results:**

Adaptors exhibited tolerance to ketoconazole concentrations up to 16 μg/mL, a significant increase from the parent strain’s inhibition at 0.015 μg/mL. All tolerant adaptors showed amplification of chromosome R, with 29 adaptors having trisomy and one having tetrasomy. This aneuploidy was unstable, reverting to euploidy and losing tolerance in drug-free conditions. Both Hsp90 and calcineurin were essential for maintaining and developing ketoconazole tolerance. Inhibition of these proteins resulted in loss of tolerance. The efflux gene *CDR1* was not required for the development of tolerance. Chromosome R trisomy and tetrasomy induce cross-tolerance to other azole antifungal agents, including clotrimazole and miconazole, but not to other antifungal classes, such as echinocandins and pyrimidines, exemplified by caspofungin and 5-flucytosine.

**Conclusion:**

Ketoconazole tolerance in *C. albicans* is mediated by chromosomal aneuploidy, specifically chromosome R amplification, and requires Hsp90 and calcineurin. These findings highlight potential targets for therapeutic intervention to combat antifungal tolerance and improve treatment outcomes.

## Introduction

The global population is witnessing a marked surge in individuals with compromised immune systems, leaving them increasingly vulnerable to infections and diseases. A complex array of factors, including demographic shifts, medical interventions, infectious diseases, genetic and autoimmune disorders, and nutritional deficiencies, is driving this trend. Consequently, the prevalence of immunocompromised individuals is escalating, and opportunistic fungal infections are becoming increasingly alarming (Fisher et al., 2022). Species from the genera *Candida*, *Cryptococcus*, and A*spergillus* are the leading causative agents of systemic fungal infections (Malcolm and Chin-Hong, 2013). Among them, *Candida albicans* is the most prevalent fungal pathogen of humans, capable of causing infections that range from superficial mucosal infections to life-threatening systemic diseases (Pappas et al., 2018).

Currently, only three classes of antifungal agents are clinically available for therapeutic use: azoles, polyenes, and echinocandins. Azoles exert their antifungal effects by inhibiting cytochrome P450 lanosterol 14-α-demethylase, thereby blocking ergosterol biosynthesis and causing the accumulation of a toxic sterol produced by the Δ-56-desaturase (Akins, 2005). Polyenes interact with ergosterol to form an ion channel-like complex, leading to the leakage of ions and small organic molecules from the cell and ultimately resulting in cell death (Kristanc et al., 2019). Echinocandins, on the other hand, inhibit the synthesis of β-(1,3)-D-glucan via noncompetitive inhibition of β-(1,3)-glucan synthase, disrupting cell wall integrity and causing severe cell wall stress (Sucher et al., 2009).

Compounding the limited antifungal arsenal is the rising incidence of antifungal resistance. The World Health Organization has highlighted antifungal resistance as a critical public health issue, emphasizing the need for research and development in this area (World Health Organization, 2022).

In contrast to resistance, which describes the ability of fungi to grow above the minimal inhibitory concentration (MIC) of the drug, antifungal tolerance refers to a transient and reversible phenomenon where a subpopulation of cells within a susceptible strain can survive and grow at drug concentrations that inhibit the majority of the population. Tolerance does not involve stable genetic mutations but rather adaptive responses that allow cells to withstand temporary exposure to antifungal agents. This can lead to persistent infections and treatment failures, as tolerant cells can repopulate once drug levels decrease [reviewed in Berman and Krysan (2020) and Yang and Berman (2024)].

Tolerance to azoles can be assessed through disk diffusion assays (DDAs) (Gerstein et al., 2016; Rosenberg et al., 2018; Xu et al., 2021; Yang et al., 2023) or broth microdilution assays (Kukurudz et al., 2022; Todd et al., 2023). In DDAs, photographs of the plates are processed using the *diskImageR* pipeline to evaluate the results. The radius of inhibition (RAD) is used to determine the level of drug resistance, while tolerance is measured by the fraction of growth (FoG) within the inhibition zone. Typically, 20% inhibition (RAD_20_ and FoG_20_) is utilized to quantify resistance and tolerance, respectively (Gerstein et al., 2016).

Azoles, particularly triazoles, are the most widely used class of antifungal agents due to their broad spectrum, high efficiency, good bioavailability, and safety profile. Antifungal resistance in *C. albicans* has been extensively documented, often resulting from mutations that enhance efflux pump activity or alter the drug target enzyme [reviewed in Lee et al. (2023)]. However, the concept of antifungal tolerance has garnered increasing attention [reviewed in Berman and Krysan (2020) and Yang and Berman (2024)].

Recent studies indicate *C. albicans* exhibits intrinsic tolerance to both triazoles (Rosenberg et al., 2018; Yang et al., 2023) and imidazoles (Xu et al., 2021), and adaptation to high concentrations of triazole drugs, such as fluconazole and posaconazole, induces the development of tolerance, not resistance, in *C. albicans* (Kukurudz et al., 2022; Todd et al., 2023; Yang et al., 2023). Despite this growing body of research on triazoles, the mechanisms underlying tolerance to imidazoles, such as ketoconazole, remain poorly understood.

**Figure 1:**
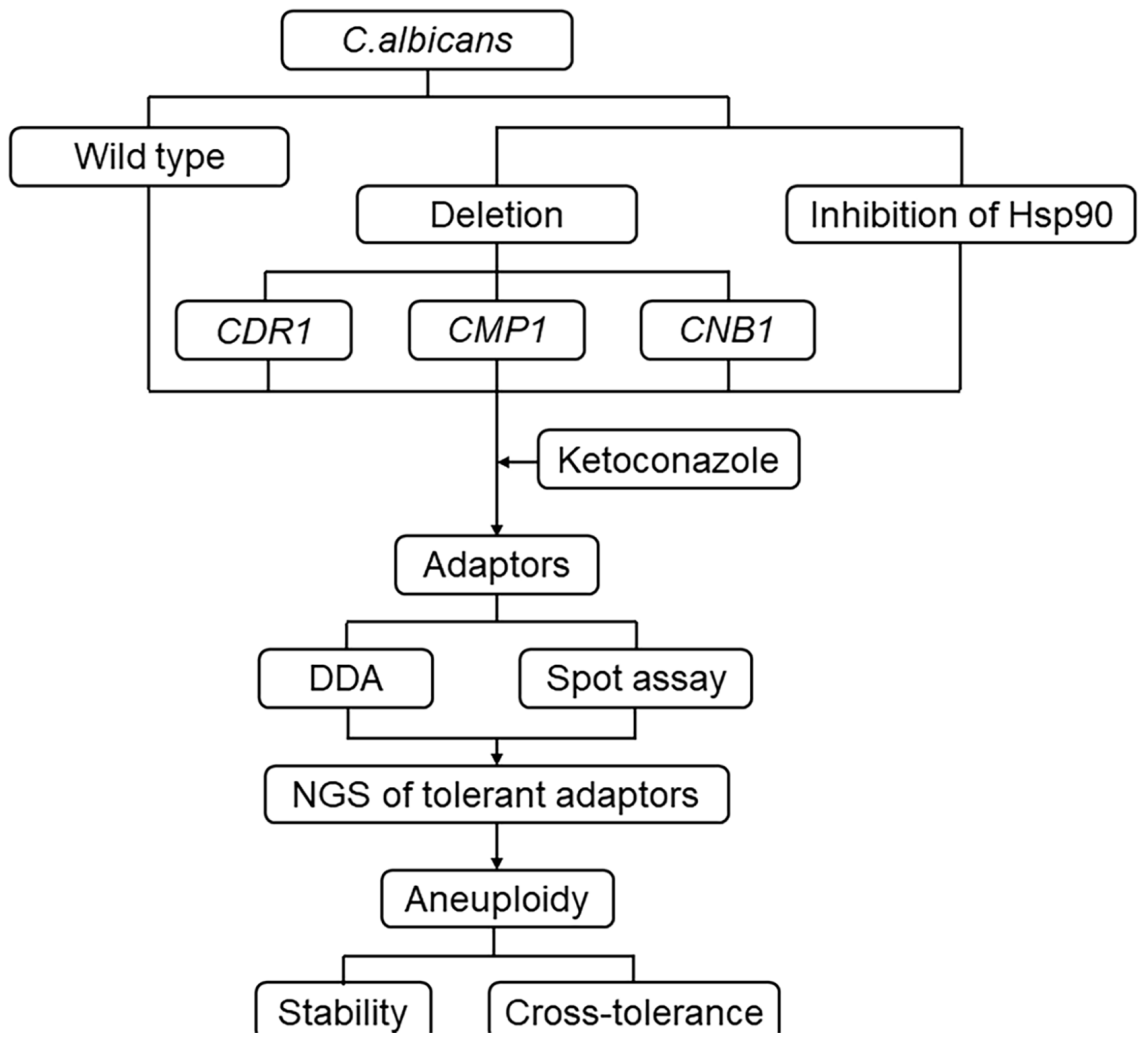
Flow chart of the study design. *C. albicans* reference strain SC5314 was exposed to a variety of KCZ concentrations. The tolerance and resistance of the strain were evaluated using a disk diffusion assay. A spot assay was also performed to assess the extent of tolerance. The tolerant adaptors were randomly sequenced, and aneuploids were tested for stability and cross-tolerance to other antifungal agents. Additionally, the requirement of Hsp90 and calcineurin, as well as the major efflux gene *CDR1*, for the development of tolerance was assayed.

This study aims to investigate the mechanisms underlying the evolution of tolerance to the imidazole drug ketoconazole (KCZ) in *C. albicans*. KCZ, introduced as the first azole-based oral treatment for systemic fungal infections in the early 1980s, serves as an important model for studying imidazole tolerance. By exposing a wild-type strain to varying concentrations of KCZ and analyzing the resulting adaptors, we seek to elucidate the genetic and molecular basis of tolerance. Specifically, we focus on the roles of chromosomal aneuploidy, Hsp90, calcineurin and efflux gene *CDR1* in this adaptive process (Figure 1). Understanding these mechanisms is crucial for developing strategies to overcome antifungal tolerance and improve clinical outcomes in the treatment of *C. albicans* infections.

## Materials and methods

### Strains and media

Strains used in this study are indicated in Supplementary Table S1. The reference strain *C. albicans* strain SC5314 was used as the wild type. Yeast extract peptone dextrose (YPD) medium was prepared with 1% yeast extract, 2% peptone, and 2% dextrose. YPD plates were prepared by adding 2% agar to the medium. KCZ (HY-B0105), NVP-HSP990 (HY-15190), Cyclosporin A (HY-B0579), caspofungin (HY-17006) and 5-flucytosine (HY-B0139) were purchased from MedChemExpress (Shanghai, China). Drugs were dissolved in dimethyl sulfoxide (DMSO) to prepare stock solutions of 10 mg/mL.

### Selection of SC5314 derived ketoconazole-tolerant adaptors

SC5314 was streaked from −80°C stock onto YPD agar. After incubation at 30°C for 1 day, several colonies were chosen randomly and suspended in distilled water. Cell density was determined using a hemocytometer. Cells were adjusted to 1 × 10^7^ cells/mL. One hundred microliters of the culture were spread on YPD plates containing KCZ at concentrations ranging from 0.015 to 32 μg/mL. Plates were incubated at 30°C for 3 days. Colonies growing on plates with 0.03 to 16 μg/mL KCZ were selected for further analysis. Eight colonies (adaptors) were randomly picked from each plate for subsequent experiments.

### Disk diffusion assay

Disk diffusion assays were conducted as previously described (Xu et al., 2021). Strains were grown on YPD agar plates at 30°C, and cell density was adjusted to 1 × 10^6^ cells/mL. An amount of 100 μL of cell suspension was plated on each plate. On each petri plate, one filter disk (GE Healthcare, United States) supplemented with 50 μg KCZ was placed in the center. The plates were incubated at 30°C for 24 h to measure the radius of inhibition at 20% (RAD_20_) and for 48 h to measure the fraction of growth at 20% (FoG_20_). Zones of inhibition (ZOI) were measured, and any colonies within the ZOI were recorded. The RAD_20_ (indicator of resistance) and FoG_20_ (indicator of tolerance) values were quantified using the *diskImageR* script (Gerstein et al., 2016).

### Spot assay

Spot assays were conducted to determine the extent of KCZ tolerance. Strains were grown on YPD agar plates at 30°C, and cell density was adjusted to 1 × 10^6^ cells/mL. Three microliters of cell cultures were spotted onto YPD plates containing various concentrations of KCZ (0.008 to 32 μg/mL). Plates were incubated at 30°C for 48 h before photographed.

### Whole-genome sequencing

Cells were grown on YPD plates at 30°C at a density of approximately 300 colonies per plate. When colonies showed different sizes on the plates, only small colonies were collected. Colonies were dispersed in 1 mL distilled water and collected by centrifugation in a microfuge for 1 min. Genomic DNA was extracted using the phenol–chloroform method (Selmecki et al., 2015). Genomic DNA library preparation were performed as described previously (Zheng et al., 2023). Briefly, genomic DNA (1 μg) was randomly fragmented with a Covaris LE220. An Agencourt AMPure XP-Medium kit was used to select, end repair, and 3′ adenylate fragments with average size of 300–400 bp, and to ligate adaptors to the ends of the 3′ adenylated fragment. The products were amplified by PCR and purified. The purified double-stranded PCR products were heat denatured and circularized with a splint oligo sequence. The single-strand circular DNA (ssCirDNA) was formatted as the final library and qualified by QC. The final qualified libraries were sequenced by BGISEQ-500. ssCir DNA molecules formed a DNA nanoball (DNB) containing more than 300 copies through rolling-cycle replication. The DNBs were loaded into a patterned nano array by using high-density DNA nano chip technology and were sequenced on the BGISEQ-500 platform using BGISEQ-500 high-throughput sequencing kit (PE100). Finally, pair-end 100 bp reads were obtained by combinational probe-anchor synthesis. Raw fastq files were uploaded to YMAP (version 1.0)^1^ (Abbey et al., 2014). Read depth was plotted as a function of chromosome position using the Assembly 22 version of the SC5314 reference genome.^2^

### Stability of aneuploids

The stability assay for aneuploids was conducted as previously described (Yang et al., 2021d). Approximately 100 cells of one chromosome R trisomy adaptor were spread on YPD plate. The plate was incubated at 30°C for 24 h. Colony size variations were observed, and representative large (L) and small (S) colonies were selected. These colonies were tested for KCZ tolerance using a disk diffusion assay and sequenced to determine their karyotypes.

### Impact of Hsp90 and calcineurin inhibitors on aneuploidy-mediated tolerance

To investigate the roles of Hsp90 and calcineurin in KCZ tolerance, disk diffusion assays were performed using YPD plates supplemented with either the Hsp90 inhibitor NVP-HSP990 (2 μg/mL) or the calcineurin inhibitor cyclosporin A (1 μg/mL). Adaptors with chromosome R trisomy and tetrasomy were tested. Strains were grown on YPD agar plates at 30°C, and cell density was adjusted to 1 × 10^6^ cells/mL. An amount of 100 μL of cell suspension was plated on each plate. Disks contained 50 μg KCZ. Plates were incubated at 30°C for 48 h, and ZOI were measured.

### Obtaining ketoconazole adaptors from gene deletion strains

The *cdr1 Δ/Δ*, *cmp1 Δ/Δ*, and *cnb1 Δ/Δ* strains were used to assess the requirement of efflux pumps and calcineurin in the development of KCZ tolerance. Construction of the deletion strains was described previously (Xu et al., 2021). Strains were grown on YPD agar plates at 30°C, and cell density was adjusted to 1 × 10^7^ cells/mL. One hundred microliters of cell cultures were spread on YPD plates containing a range of KCZ concentrations (0.002 to 4 μg/mL). The plates were incubated at 30°C for 3 days. Adaptors were randomly selected and tested for KCZ tolerance using disk diffusion and spot assays.

### Data analysis

RAD_20_ and FoG_20_ values were calculated using the *diskImageR* script to quantify resistance and tolerance, respectively. Student’s *t*-test was performed using GraphPad Prism software. Differences were considered statistically significant at *p* < 0.05.

## Results

### Exposure of SC5314 to high amount of ketoconazole selects tolerant adaptors

**Figure 2:**
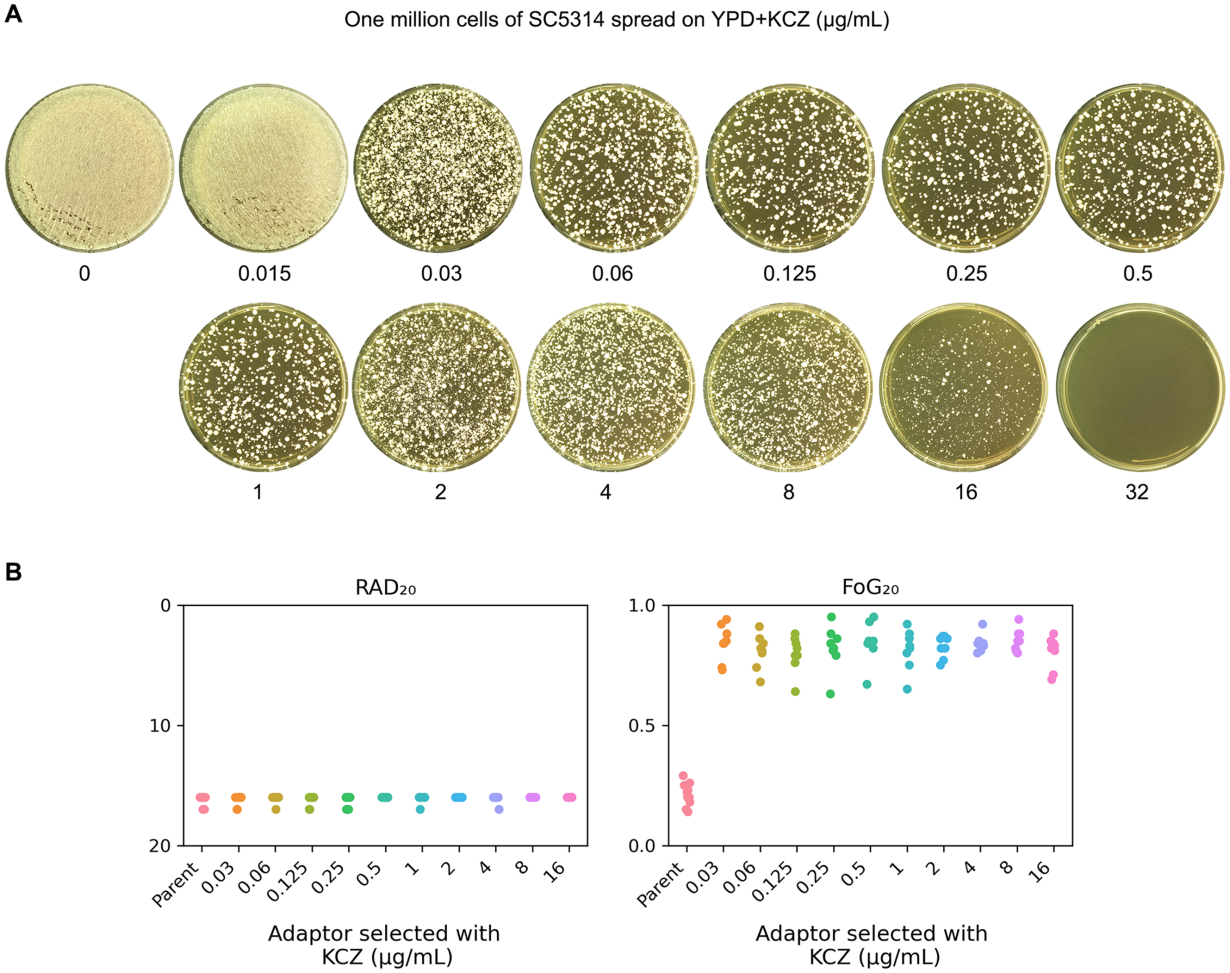
Exposure of SC5314 to ketoconazole selects for tolerance. **(A)** Approximately one million cells of *C. albicans* lab strain SC5314 were spread on YPD plates supplemented with KCZ. Drug concentrations are indicated in the figure. The plates were incubated at 30°C for 3 days, then photographed. **(B)** Eight adaptors from each drug plate (0.03–16 μg/mL KCZ) were randomly selected and tested with a disk diffusion assay. Disks contained 50 μg KCZ. For the parent, 10 individual colonies were tested as 10 biological replicates. RAD_20_ and FoG_20_ values were generated using the *diskImageR* script.

To obtain KCZ-selected adaptors, approximately one million cells of SC5314 were spread on YPD plates containing 0.015–32 μg/mL KCZ. After incubation at 30°C for 3 days, the plate containing 0.015 μg/mL KCZ exhibited lawn growth, whereas the plate with 32 μg/mL KCZ showed no growth. On the plates with 0.03–16 μg/mL KCZ, several hundred colonies were visibly present (Figure 2A). Eight colonies (hereafter referred to as adaptors) were randomly selected from each plate for further analysis.

DDAs were performed to determine whether the adaptors exhibited resistance or tolerance to KCZ. Disks containing 50 μg of KCZ were used. The parent strain exhibited a clear ZOI. In contrast, all adaptors displayed colonies growing within the ZOI (Supplementary Figure S1). RAD_20_ and FoG_20_ were quantified using the *diskImageR* script. The parent strain had RAD_20_ and FoG_20_ values of 16.20 ± 0.42 and 0.21 ± 0.05, respectively. In contrast, the adaptors exhibited RAD_20_ and FoG_20_ values of 16.13 ± 0.33 and 0.83 ± 0.07, respectively (Figure 2B). There was no significant difference of RAD_20_ values between the parent and the adaptors (*p* > 0.05, student’s *t*-test), but the adaptors exhibited significantly higher FoG_20_ levels than parent (*p* < 0.001, student’s *t*-test).

### Adaptors derived from different selection force have similar extent of tolerance

**Figure 3:**
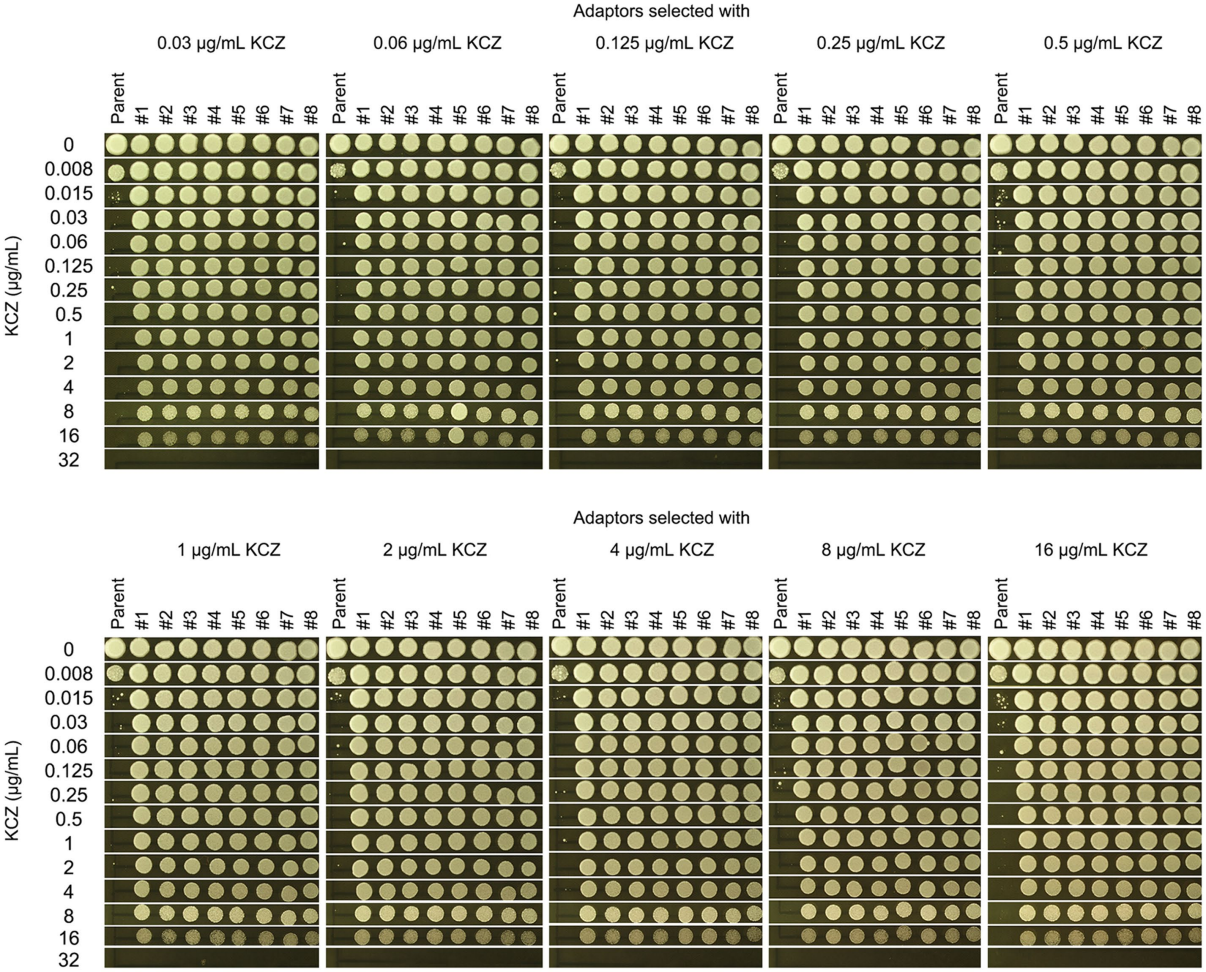
Measurement of the level of ketoconazole tolerance in adaptors. The parent SC5314 was compared to the KCZ-selected adaptors for the extent of tolerance. A spot assay was performed on YPD plates supplemented with 0.008–32 μg/mL KCZ. For each strain, cells were adjusted to 1 × 10^6^ cells/mL, and 3 μL of cell suspensions were spotted on the plates. The plates were incubated at 30°C for 2 days, then photographed.

To determine the extent of KCZ tolerance in the adaptors, a spot assay was performed using a wide range of KCZ concentrations, from 0.008 to 32 μg/mL. The parent strain and all the adaptors were tested by spotting 3 μL of cell suspensions at a density of 1.0 × 10^6^ cells/mL onto drug-containing plates. As shown in Figure 3, the growth of the parent strain was completely inhibited at 0.015 μg/mL, whereas all adaptors were capable of growth at 16 μg/mL, and none could grow at 32 μg/mL. Thus, all adaptors exhibited a similar extent of KCZ tolerance, which was at least 1,067-fold higher than that of the parent strain.

### All adaptors have amplification of chromosome R

**Figure 4:**
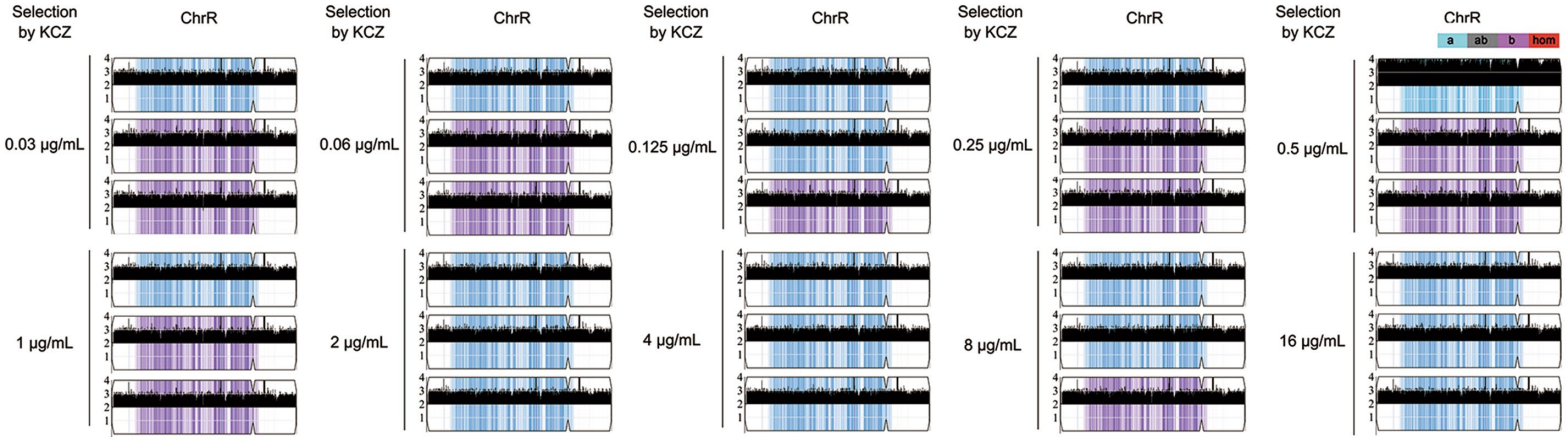
Copy number of chromosome R in adaptors. Randomly three adaptors from each drug plate were sequenced. The sources of the adaptors are indicated in the figure. Shown is the copy number of chromosome R. *Y*-axis indicated copy number of the sequenced fragments. *X*-axis indicates the loci of the fragments on chromosome R. Homologs A and B are indicated by cyan and magenta colors, respectively. The plot was generated using Ymap.

As described above, 9 adaptors were randomly selected from each drug plate. From each group, 3 adaptors were randomly chosen for sequencing. In total, 30 adaptors were subjected to whole-genome sequencing. Our analysis revealed that all adaptors had amplifications of chromosome R (ChrR): 29 had three copies and 1 had four copies. Notably, there was no biased duplication of ChrR homologs. Among the ChrR trisomy adaptors, 17 and 12 adaptors had duplications of the A and B homologs, respectively (Figure 4). There was no whole chromosomal or segmental aneuploidy of other chromosomes (Supplementary Figure S2).

### Tolerance is controlled by chromosome R copy number

**Figure 5:**
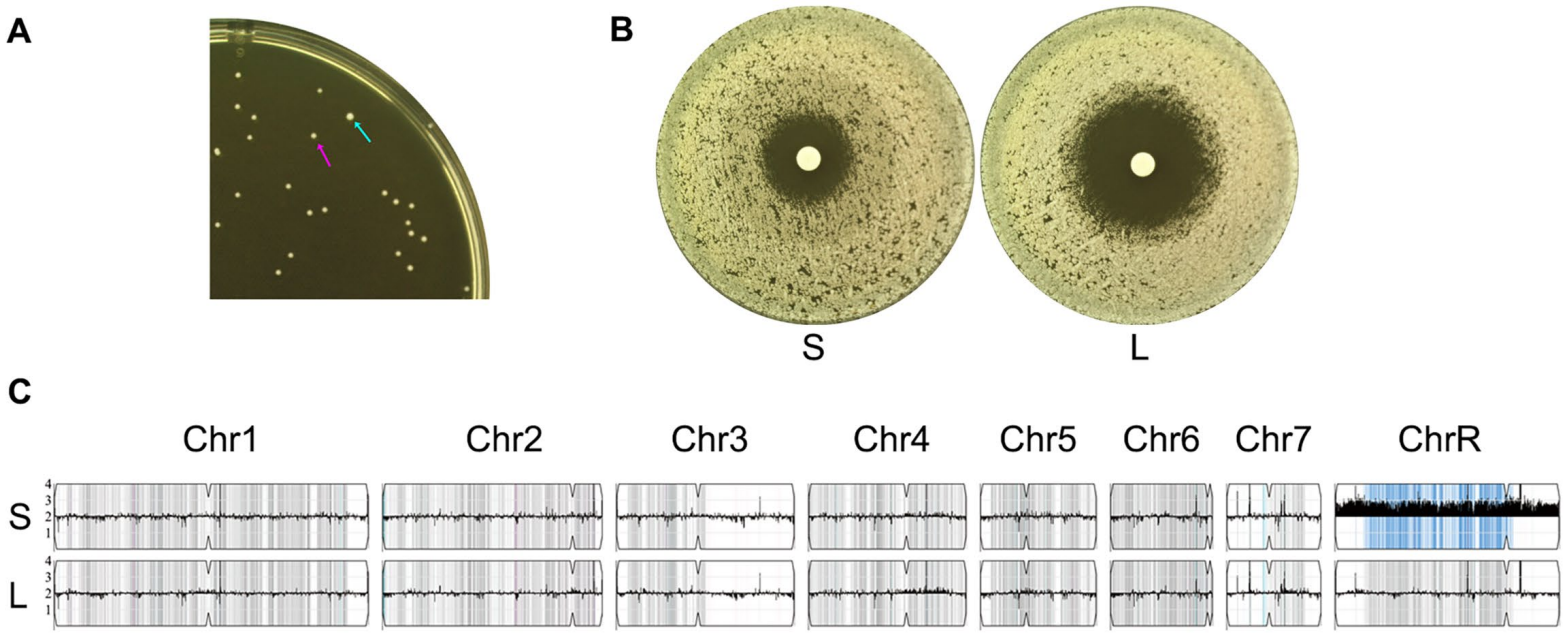
Phenotypic and genomic instability of chromosome R trisomy. **(A)** Approximately 100 cells of an adaptor with chromosome R trisomy were spread on YPD plates. The plate was incubated at 30°C for 24 h, then photographed. The cyan arrow indicates the large colony, while the magenta arrow indicates a small colony. **(B)** A disk diffusion assay was performed using disks containing 50 μg KCZ. “S” and “L” denote small and large colonies picked from the plate shown in **A**. **(C)** Whole-genome sequencing of “S” and “L” colonies. The karyotypes were visualized using Ymap.

Aneuploidy typically confers a fitness loss. In the absence of selective pressure, aneuploidy is usually unstable and spontaneously reverts to euploidy (Yang et al., 2021d). We investigated whether the ChrR trisomy was also phenotypically and genomically unstable. Cells from one ChrR trisomy adaptor (#1 from the 0.03 μg/mL KCZ plate) were spread on a YPD plate. After 24 h of growth at 30°C, colony size variations were observed: one colony (indicated by a cyan arrow in Figure 5A, hereafter referred to as the L colony) was significantly larger than the other colonies (hereafter referred to as S colonies). The L colony and one randomly selected S colony (indicated by a magenta arrow in Figure 5A) were tested using a disk diffusion assay for KCZ tolerance. The S colony exhibited growth inside the ZOI, whereas the L colony had a clear ZOI (Figure 5B). Both colonies were sequenced. The S colony retained the same karyotype as the progenitor, which was ChrR trisomy. In contrast, the L colony was euploid (Figure 5C). We propose that the S colony with 3 copies of ChrR (AAB) spontaneously lost one extra copy and reverted to diploidy (AB for ChrR). Taken together, our results suggest that in the absence of stress, ChrR trisomy spontaneously reverts to euploidy, concomitantly losing KCZ tolerance.

### Chromosome R trisomy mediated tolerance is dependent on Hsp90 and calcineurin

Hsp90 (Heat Shock Protein 90) is a molecular chaperone that plays key cellular roles by eliciting molecular response to environmental changes, morphogenesis, antifungal resistance, and fungal pathogenicity. It interacts with a range of client proteins, including calcineurin, which are involved in various cellular processes, such as signaling pathways, gene expression, and stress responses, which are critical for antifungal resistance (Cowen et al., 2009). Calcineurin is a calcium-dependent serine/threonine phosphatase. It plays a critical role in antifungal resistance by regulating transcription factors, efflux pumps, cell wall integrity, and responses to oxidative stress (Juvvadi et al., 2017).

**Figure 6:**
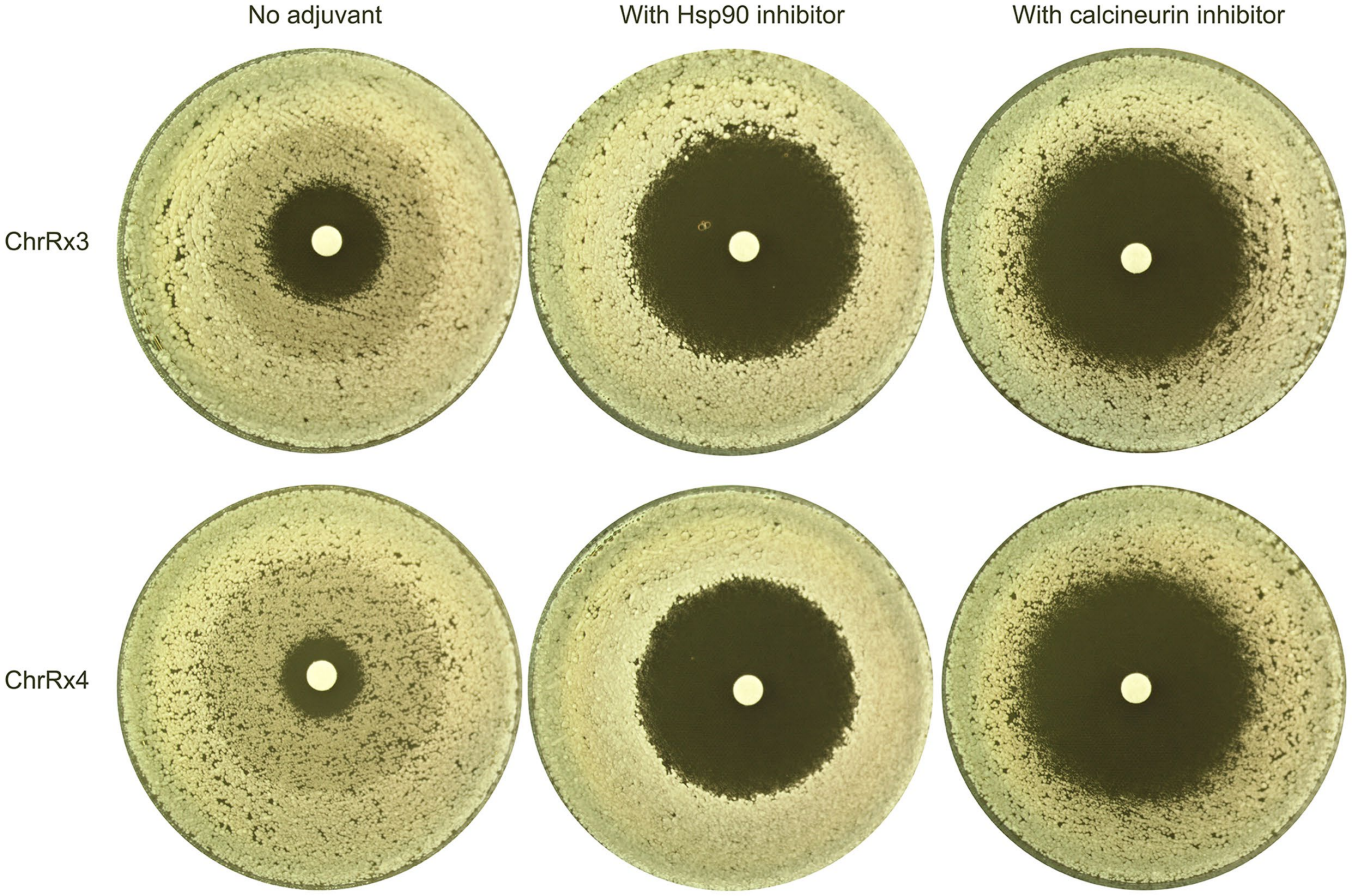
Requirement of Hsp90 and calcineurin for maintenance of ketoconazole tolerance in chromosome R trisomy and tetrasomy strains. Two adaptors bearing chromosome R trisomy and tetrasomy, respectively, were tested with disk diffusion assays using inhibitors of Hsp90 and calcineurin. The disks contained 50 μg KCZ. The Hsp90 inhibitor used was NVP-HSP990, with a final concentration of 2 μg/mL in the plate. The calcineurin inhibitor was cyclosporin A, with a final concentration of 1 μg/mL. The plates were incubated at 30°C for 48 h, then photographed.

Previously, we demonstrated that KCZ tolerance in the wild-type strain SC5314 is dependent on Hsp90 and calcineurin (Xu et al., 2021). Here, we investigated whether these two proteins are also required for tolerance mediated by aneuploidy. One adaptor with ChrR trisomy and one adaptor with ChrR tetrasomy were tested. A disk diffusion assay was performed using YPD medium, as well as YPD medium supplemented with the Hsp90 inhibitor NVP-HSP990 (Menezes et al., 2012) or the calcineurin inhibitor cyclosporin A (Williams and Gooch, 2012). The disks contained 50 μg of KCZ. Notably, both strains exhibited clear ZOI on plates with inhibitors of Hsp90 and calcineurin (Figure 6). Thus, our results indicate that both Hsp90 and calcineurin are essential for maintaining KCZ tolerance in ChrR trisomy and tetrasomy strains.

### Hsp90 is required for development of ketoconazole tolerance and resistance

**Figure 7:**
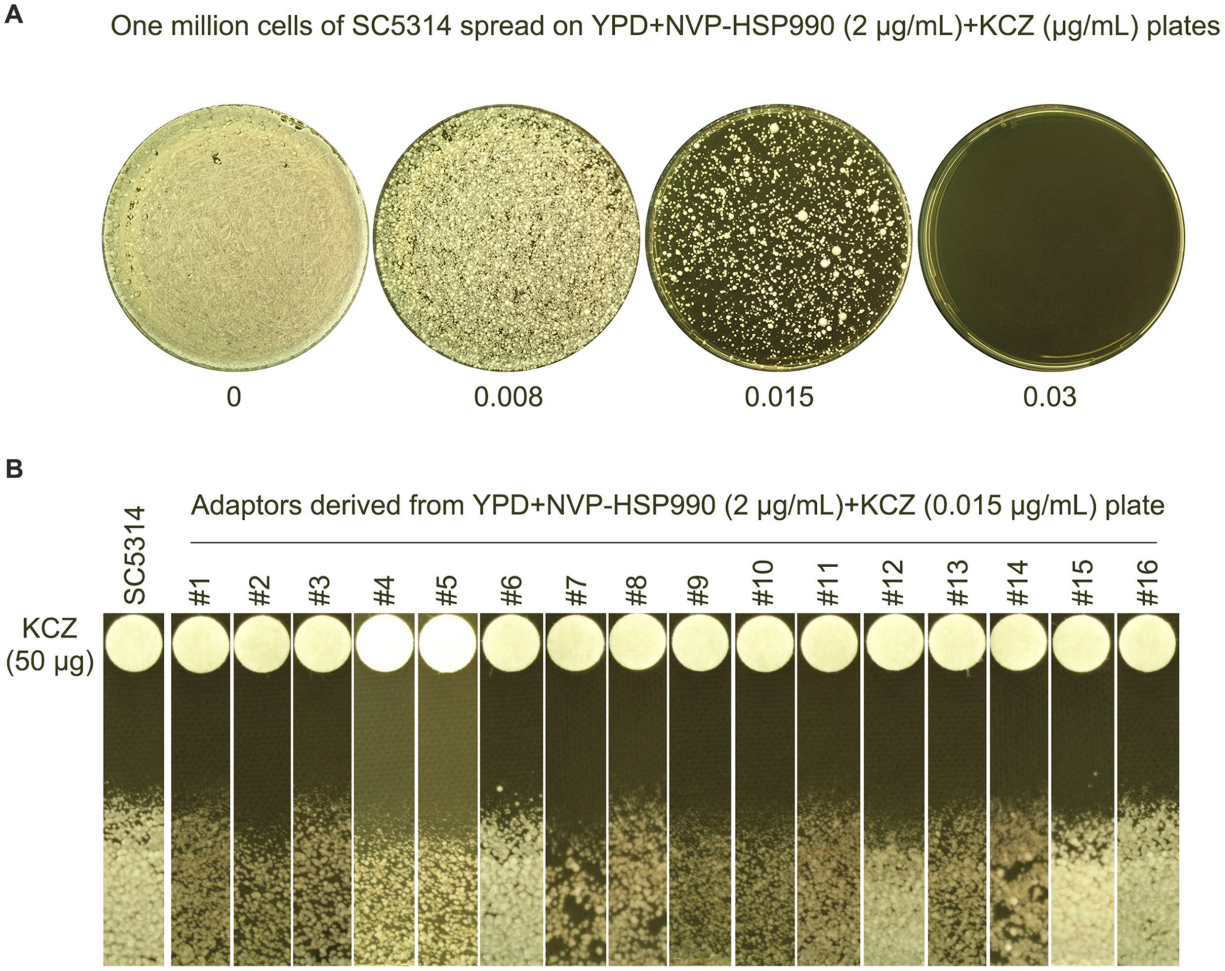
Requirement of Hsp90 for evolution of ketoconazole tolerance. **(A)** Approximately of wild-type strain SC5314 were spread on YPD plate supplemented with Hsp90 inhibitor NVP-HSP990 and KCZ. Drug concentrations are indicated in the figure. The plates were incubated at 30C for 3 days, then photographed. **(B)** Randomly 16 colonies (#1–#16) were chosen from the plate containing 0.015 μg/mL KCZ. Disk diffusion assay was performed using disks containing 50 μg KCZ. The plates were incubated at 30C for 2 days, then photographed.

Next, we investigated whether Hsp90 was required for the development of KCZ tolerance. The wild-type strain SC5314 was spread on a YPD plate supplemented with the Hsp90 inhibitor NVP-HSP990 as well as KCZ (Figure 7A). Randomly 16 adaptors that appeared on the YPD + 0.015 μg/mL KCZ plate were tested. A disk diffusion assay revealed that all adaptors had clear ZOI, and there was no apparent change in the size of the ZOI (Figure 7B), indicating that the colonies were neither tolerant nor resistant. Thus, our results demonstrate that Hsp90 is essential for the development of KCZ tolerance and resistance.

### Calcineurin is required for development of ketoconazole tolerance and resistance

**Figure 8:**
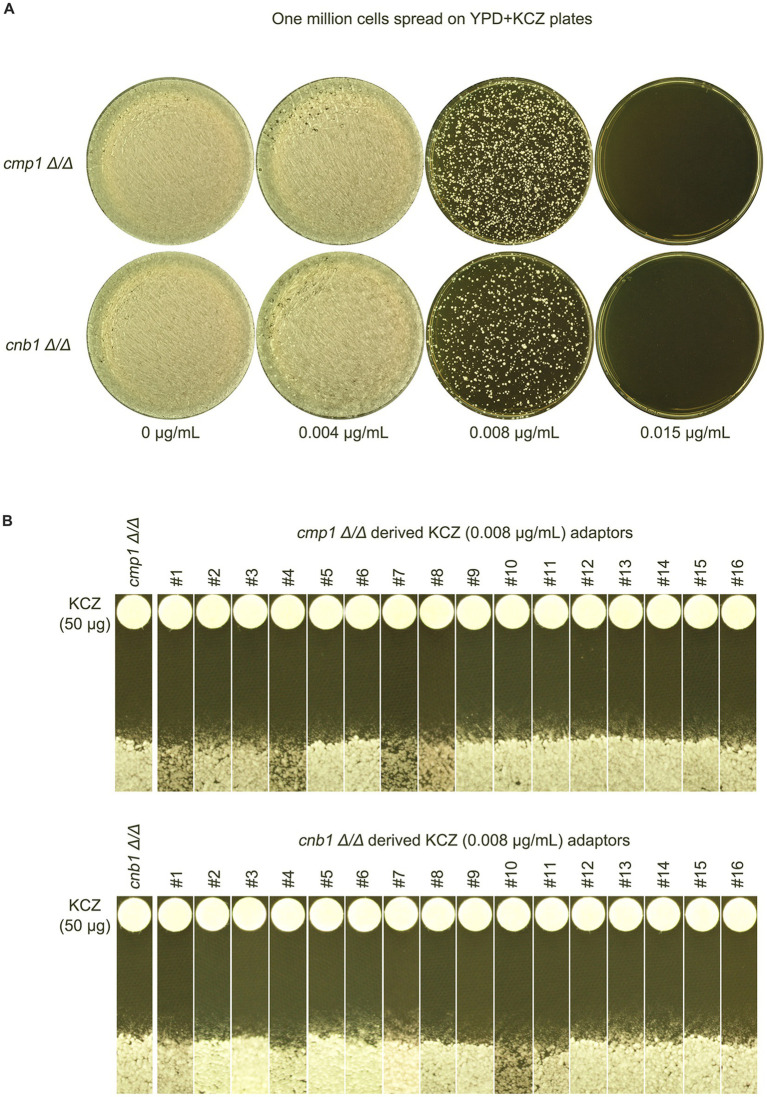
Requirement of calcineurin subunits for the evolution of ketoconazole tolerance. **(A)** Deletion strains of *CMP1* and *CNB1*, encoding the catalytic and regulatory subunits of calcineurin, respectively, were derived from SC5314. These strains were spread on YPD plates supplemented with KCZ. Drug concentrations are indicated in the figure. The plates were incubated at 30°C for 3 days, then photographed. **(B)** Sixteen colonies were randomly chosen from each plate with 0.008 μg/mL KCZ. A disk diffusion assay was performed to measure KCZ tolerance, using disks containing 50 μg KCZ. The plates were incubated at 30°C for 2 days, then photographed.

Calcineurin is a complex consisting of a catalytic subunit A and a regulatory subunit B, which are encoded by *CMP1* and *CNB1*, respectively, in the *C. albicans* genome. To investigate whether calcineurin is required for the development of KCZ tolerance, *cmp1 Δ/Δ* and *cnb1 Δ/Δ* strains were spread on YPD plates containing KCZ (Figure 8A). Sixteen adaptors derived from each deletion strain were randomly selected. A disk diffusion assay was performed, and all adaptors on the plate with KCZ disks exhibited clear ZOI, with no obvious change in the size of the ZOI, indicating that they were neither tolerant nor resistant to KCZ (Figure 8B). Thus, our results demonstrate that both subunits of calcineurin are essential for the development of KCZ tolerance and resistance.

### *CDR1* is not required for development of ketoconazole tolerance

In the *C. albicans* genome, *CDR1*, *CDR2*, and *MDR1* are the major efflux genes that play a crucial role in antifungal resistance. Previously, we found *CDR1* was partially required for maintaining KCZ tolerance in wild-type strains. Here, we investigated whether *CDR1* was required for the development of KCZ tolerance.

**Figure 9:**
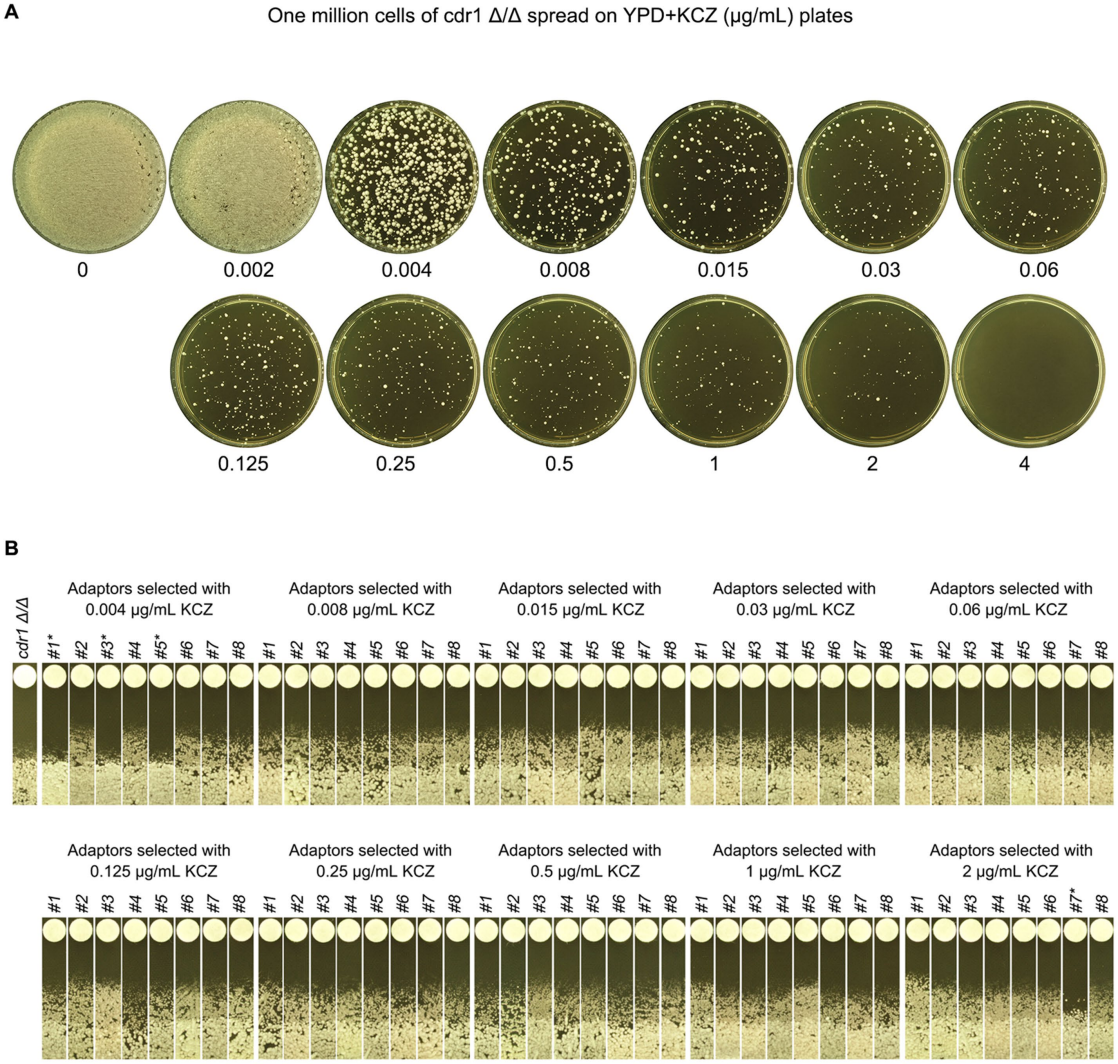
Requirement of *CDR1* for the evolution of ketoconazole tolerance. **(A)** *CDR1* deletion strain was derived from SC5314. Approximately one million cells of the deletion strain were spread on YPD plates supplemented with KCZ at the indicated concentrations. The plates were incubated at 30°C for 3 days and then photographed. **(B)** Eight colonies were randomly selected from the plates with 0.004–2 μg/mL KCZ. A disk diffusion assay was performed using disks containing 50 μg KCZ. The sources of the adaptors are indicated. * indicates non-tolerant adaptors. The plates were incubated at 30°C for 2 days and then photographed.

**Figure 10:**
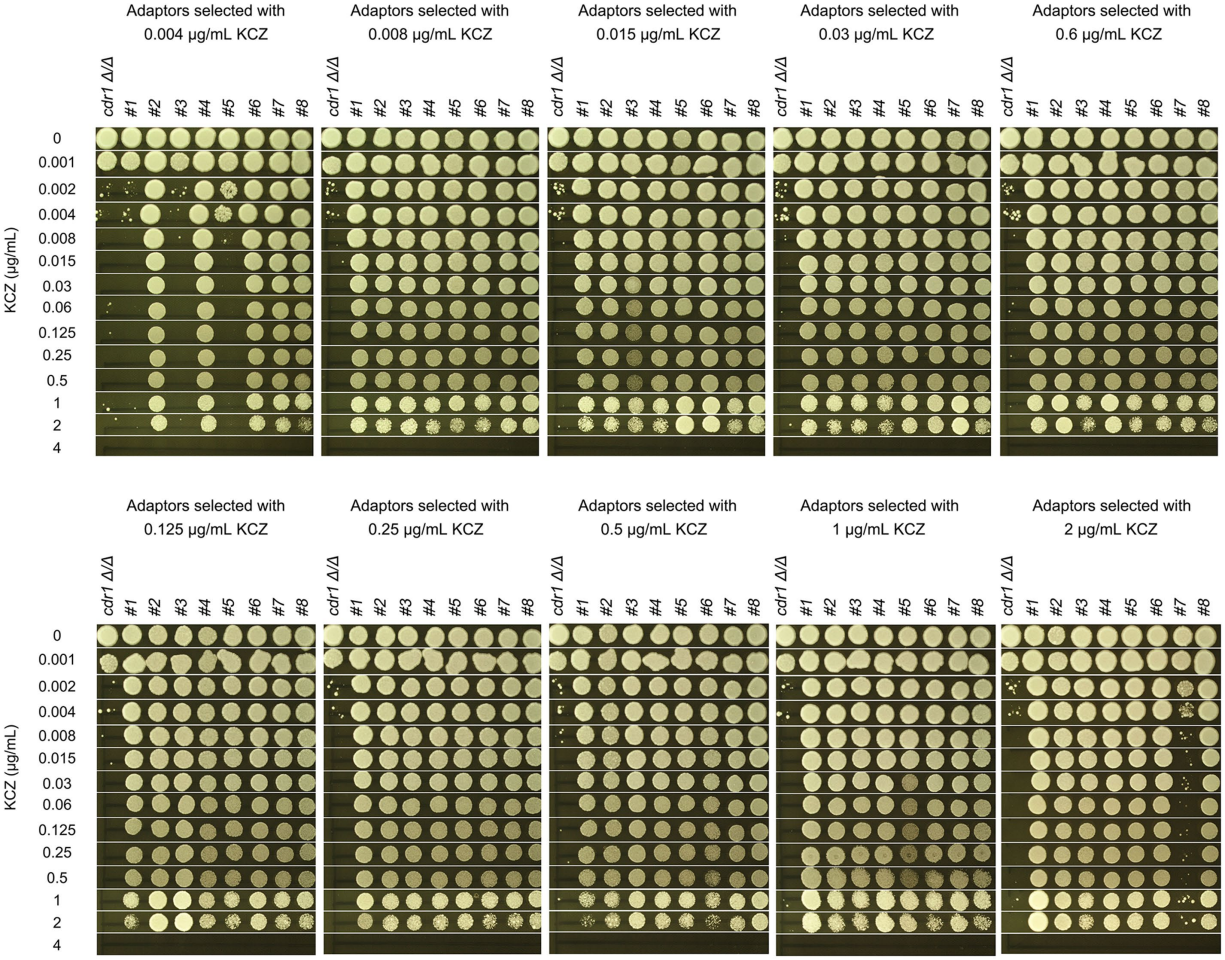
Assessment of ketoconazole tolerance in *cdr1 Δ/Δ* derived adaptors. All adaptors derived from the *CDR1* deletion strain were compared to the parent strain for their level of KCZ tolerance. The measurement was performed using YPD plates containing 0.001–4 μg/mL KCZ. For each strain, cells were adjusted to 1 × 10^6^ cells/mL, and 3 μL of cell suspensions were spotted onto the plates. The plates were incubated at 30°C for 2 days and then photographed.

To this end, *cdr1 Δ/Δ* strain was spread on YPD plate supplemented with a wide range of KCZ concentrations. On the plate with 0.002 μg/mL, there was lawn growth of colonies. On the plate with 4 μg/mL, there were no colonies. On the plates with 0.004–2 μg/mL KCZ, a few hundred of colonies were obviously visible (Figure 9A). Randomly 8 adaptors from each plate were chosen. To investigate whether the adaptors acquired KCZ tolerance, disk diffusion assay was performed with disks containing 50 μg KCZ. Among the total 80 adaptors, 76 had colonies growing inside ZOI. Only 4 adaptors were had clear ZOI: 3 and 1 adaptors derived from 0.004 and 2 μg/mL KCZ plates, respectively. None of them had apparent change of size of ZOI (Figure 9B). Spot assay was performed to compare extent of tolerance. Growth of *cdr1 Δ/Δ* was inhibited by 0.002 μg/mL KCZ. All the 76 tolerant adaptors could grow on the plate with 2 μg/mL KCZ but none could growth on the plate with 4 μg/mL KCZ. Among the 4 non-tolerant adaptors, growth of 2 adaptors was inhibited by 0.002 μg/mL KCZ, and the other 2 adaptors were inhibited by 0.008 μg/mL KCZ (Figure 10). Thus, most of the *cdr1 Δ/Δ* strain derived KCZ adaptors acquired tolerance, and they had similar level of tolerance.

### Amplification of ChrR confers cross-tolerance to azoles but not tolerance to echinocandins or pyrimidines

Previous research has shown that *C. albicans* is highly tolerant of aneuploidy. In the SC5314 strain, trisomy and tetrasomy of each of the 8 pairs of chromosomes can occur depending on the selective pressure (Yang et al., 2021d). Different stresses may select for the same aneuploidy, leading to cross-tolerance to unrelated stresses (Yang et al., 2019, 2021b). Therefore, aneuploidy induced by one stress has the potential to confer cross-tolerance to other stresses. Here, we investigated whether the trisomy and tetrasomy adaptors selected by KCZ conferred cross-tolerance to other azole antifungals and/or other classes of antifungal drugs, such as echinocandins (caspofungin) and pyrimidines (5-flucytosine).

**Figure 11:**
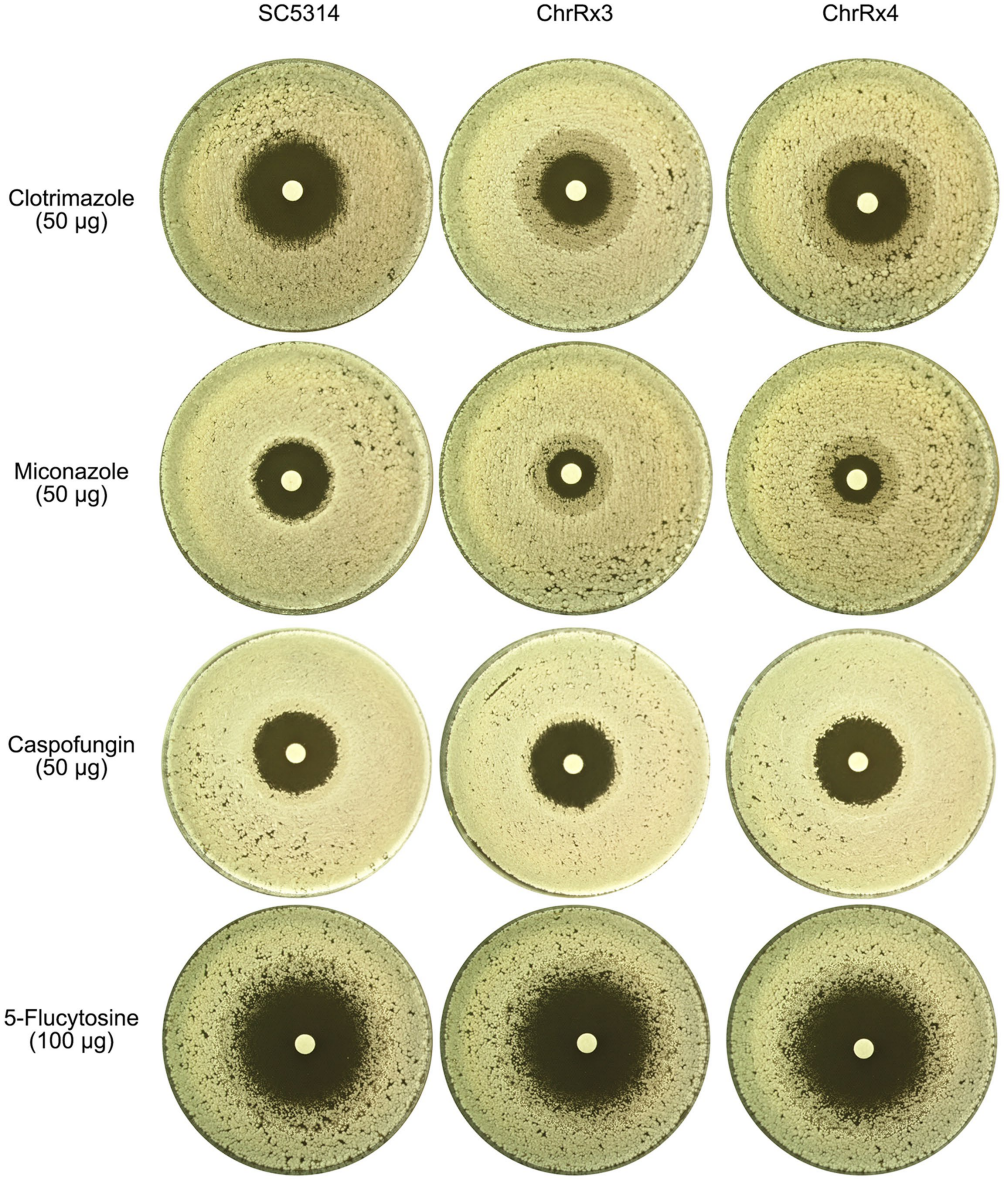
Cross-tolerance of aneuploids to different classes of antifungal drugs. The wild-type strain SC5314 and two strains bearing ChrR trisomy and tetrasomy, respectively, were evaluated for their tolerance to a range of antifungal drugs, including azoles (clotrimazole and miconazole), echinocandins (caspofungin), and pyrimidines (5-flucytosine). The concentrations of the drugs in the disks are depicted in the figure. The temperature used for testing was 37°C for 5-flucytosine and 30°C for the other drugs. The plates were incubated for 2 days before being photographed.

We tested the parent SC5314 strain, as well as one trisomy and one tetrasomy adaptor of ChrR. DDAs were performed using disks containing two azole antifungals (clotrimazole and miconazole), one echinocandin (caspofungin), and one pyrimidine (5-flucytosine). For azoles, the SC5314 strain exhibited a clear ZOI, while the trisomy and tetrasomy strains showed growth within the ZOI. For caspofungin and 5-flucytosine, all strains exhibited a clear ZOI (Figure 11). Therefore, trisomy and tetrasomy of ChrR conferred cross-tolerance to other azole antifungals but not to echinocandins or pyrimidines.

## Discussion

The results of this study provide critical insights into the mechanisms by which *C. albicans* adapts to KCZ stress. Through a series of experiments, we have demonstrated that exposure to increasing concentrations of KCZ selects for adaptors with markedly increased tolerance, without a corresponding increase in resistance. This tolerance is closely associated with whole chromosomal aneuploidy, specifically the amplification of ChrR.

The selection process revealed that *C. albicans* can adapt to a broad range of KCZ concentrations. The adaptors obtained exhibited growth at concentrations up to 16 μg/mL KCZ, which is a significant increase compared to the parent strain inhibited at 0.015 μg/mL. This finding underscores the potential for *C. albicans* to rapidly adapt to antifungal agents, posing challenges for clinical treatment. In comparison to the parent strain SC5314, the RAD_20_ values of the adaptors showed no significant difference, whereas the FoG_20_ values were significantly elevated. RAD is a measurement of resistance. It is inversely correlated with the MIC. FoG is a measurement of tolerance. It correlates with the proportion of colonies that appear inside ZOI at drug concentrations above the MIC (relative to total colonies growing in the absence of drug) [reviewed in Berman and Krysan (2020)]. Thus, the wild-type strain adapted to KCZ primarily through the acquisition of tolerance, rather than resistance.

Our genomic analysis identified ChrR amplification as a key feature in all tolerant adaptors. The prevalence of trisomy in 29 adaptors and tetrasomy in one adaptor suggests a strong selection pressure favoring aneuploidy as a mechanism of tolerance. The instability of ChrR trisomy, reverting to euploidy and concomitant loss of tolerance in the absence of KCZ, further confirms the adaptive significance of ChrR amplification under antifungal stress. These results align with previous findings that aneuploidy can confer adaptive advantages in fungi but may incur fitness costs in drug-free environments (Yang et al., 2021d).

The essential roles of Hsp90 and calcineurin in maintaining KCZ tolerance in aneuploid strains were clearly demonstrated. The inhibition of these proteins resulted in the loss of tolerance, indicating their crucial involvement in the adaptive process. This is consistent with their known functions in cellular stress responses and antifungal resistance pathways (Cowen et al., 2009; Singh et al., 2009; Robbins et al., 2011). Notably, both proteins are required not only for maintaining tolerance but also for its development, highlighting their potential as targets for therapeutic intervention to prevent or counteract antifungal tolerance.

Interestingly, our investigation into the role of the efflux gene *CDR1* revealed that it is not necessary for the development of KCZ tolerance, despite its partial requirement for maintaining it. This suggests that the mechanisms underlying the development and maintenance of tolerance can be distinct and that multiple pathways may contribute to the adaptive response. The fact that most *cdr1 Δ/Δ*-derived adaptors acquired tolerance supports the notion that *C. albicans* can utilize diverse genetic strategies to survive antifungal pressure.

Aneuploidy has a profound impact on gene regulation, directly influencing the copy number and expression of hundreds of genes on the aneuploid chromosome, while also affecting the expression of genes on euploid chromosomes through complex regulatory networks. This dual effect has the potential to result in pleiotropic phenotype changes (Pavelka et al., 2010; Chen et al., 2012). Previous studies have shown that aneuploidy can confer cross-tolerance to unrelated drugs in several pathogenic fungi, including *C. albicans*, *C. parapsilosis*, and *Cryptococcus neoformans* (Yang et al., 2019, 2021a,b,c; Sun et al., 2023). However, our findings indicate that ChrR trisomy and tetrasomy specifically confer cross-tolerance to azole drugs, but not to echinocandins or 5-flucytosine.

To the best of our knowledge, we have performed a unique study in association with the role of aneuploidy in conferring tolerance to KCZ and cross-tolerance to other azole antifungals in *C. albicans*. Through the application of high-throughput screening, we identified a panel of adaptors that exhibit tolerance to KCZ, which were derived from plates containing a wide range of drug concentrations, including concentrations that are typically inhibitory to wild-type *C. albicans* cells. The ability of *C. albicans* to rapidly develop tolerance to KCZ and cross-tolerance to other azoles through chromosomal aneuploidy and the involvement of Hsp90 and calcineurin has significant clinical implications. Antifungal treatments targeting these pathways could potentially mitigate the development of drug tolerance and improve therapeutic outcomes. Moreover, monitoring for aneuploidy in clinical isolates may provide insights into emerging tolerance and resistance patterns, informing treatment strategies.

Our study only selected 9 adaptors from each drug plate, which may not be representative of all possible adaptors that could be selected. The study does not provide a detailed mechanistic understanding of how ChrR trisomy and tetrasomy confer cross-tolerance to azole drugs. Resistance to azoles is generally attributed to altered target sites and increased efflux (Lee et al., 2023). Recent studies have shown that the maintenance of azole tolerance in wild-type strains also relies on the *CDR1* gene (Xu et al., 2021; Guo et al., 2024). Therefore, we propose that ChrR trisomy may potentiate KCZ tolerance by up-regulating the expression of genes encoding drug efflux pumps and/or the target protein Erg11, as well as other *ERG* genes involved in the ergosterol biosynthesis pathway.

Future research should explore the broader genetic landscape of KCZ tolerance in *C. albicans*, including potential contributions from other chromosomes and genes. Longitudinal studies on the stability of aneuploidy and tolerance in various environmental conditions will further elucidate the evolutionary dynamics of these adaptations. Additionally, investigating interactions between Hsp90, calcineurin, and other cellular pathways may uncover new therapeutic targets to combat antifungal tolerance and resistance.

## Conclusion

In summary, our study highlights the adaptive capacity of *C. albicans* to KCZ through chromosomal aneuploidy and the critical roles of Hsp90 and calcineurin in this process. These findings enhance our understanding of antifungal tolerance mechanisms and provide a foundation for developing strategies to counteract fungal drug tolerance.

## Data availability statement

The datasets presented in this study can be found in online repositories. The names of the repository/repositories and accession number(s) can be found in the article/Supplementary material.

## Author contributions

LZ: Data curation, Formal analysis, Investigation, Validation, Visualization, Writing – review & editing. YX: Data curation, Formal analysis, Funding acquisition, Investigation, Methodology, Writing – review & editing. CW: Investigation, Visualization, Writing – review & editing. LG: Funding acquisition, Project administration, Resources, Supervision, Writing – original draft, Writing – review & editing.

## Footnotes

^1^ http://lovelace.cs.umn.edu/Ymap/

^2^ http://www.candidagenome.org/download/sequence/C_albicans_SC5314/Assembly22/current/C_albicans_SC5314_A22_current_chromosomes.fasta.gz
