# Supplementary material for: Ketoconazole induces reversible antifungal drug tolerance mediated by trisomy of chromosome R in Candida albicans
Source: Front Microbiol. 2024 Jul 30;15:1450557. doi: 10.3389/fmicb.2024.1450557 (PMC11319258; doi:10.3389/fmicb.2024.1450557)
Supplement: Supplementary file 1 [file Data_Sheet_1.ZIP › Supplementary materials.docx]

**Supplementary materials**

**Table S1. Strains used in this study**

**Figure S1. Disk diffusion assay of SC5314-derived ketoconazole adaptors**

Eight KCZ adaptors derived from SC5314, selected from each drug plate, were tested using a disk diffusion assay with disks containing 50 μg KCZ. The sources of the adaptors are indicated in the figure. The plates were incubated at 30°C for 2 days and then photographed.

**Figure S2. Karyotypes of SC5314-derived ketoconazole adaptors**

A subset of 30 adaptors was selected from the KCZ adaptors derived from SC5314. The sources of the adaptors are indicated in the figure. The karyotypes are visualized using Ymap.
