# Supplementary material for: Ketoconazole induces reversible antifungal drug tolerance mediated by trisomy of chromosome R in Candida albicans
Source: Front Microbiol. 2024 Jul 30;15:1450557. doi: 10.3389/fmicb.2024.1450557 (PMC11319258; doi:10.3389/fmicb.2024.1450557)
Supplement: Supplementary file 1 [file Data_Sheet_1.ZIP › Table S1. Strains used in this study.docx]

Table S1. Strains used in this study

| Strain | Genotype | Parent | Source |
| --- | --- | --- | --- |
| SC5314 | Wild type |  | (Noble and Johnson, 2005) |
| YCA779 | cdr1::FRT/cdr1::NAT1 flp | SC5314 | (Xu et al., 2021) |
| YCA641 | cmp1::FRT/cmp1::NAT1 flp | SC5314 | (Xu et al., 2021) |
| YCA623 | cnb1::FRT/cnb1::NAT1 flp | SC5314 | (Xu et al., 2021) |

**References**

Noble, S.M., and Johnson, A.D. (2005). Strains and strategies for large-scale gene deletion studies of the diploid human fungal pathogen Candida albicans. *Eukaryot Cell* 4(2)**,** 298-309. doi: 10.1128/EC.4.2.298-309.2005.

Xu, Y., Lu, H., Zhu, S., Li, W.Q., Jiang, Y.Y., Berman, J., et al. (2021). Multifactorial Mechanisms of Tolerance to Ketoconazole in Candida albicans. *Microbiol Spectr* 9(1)**,** e0032121. doi: 10.1128/Spectrum.00321-21.
