# Supplementary figures and images for: Ketoconazole induces reversible antifungal drug tolerance mediated by trisomy of chromosome R in Candida albicans
Source: Front Microbiol. 2024 Jul 30;15:1450557. doi: 10.3389/fmicb.2024.1450557 (PMC11319258; doi:10.3389/fmicb.2024.1450557)

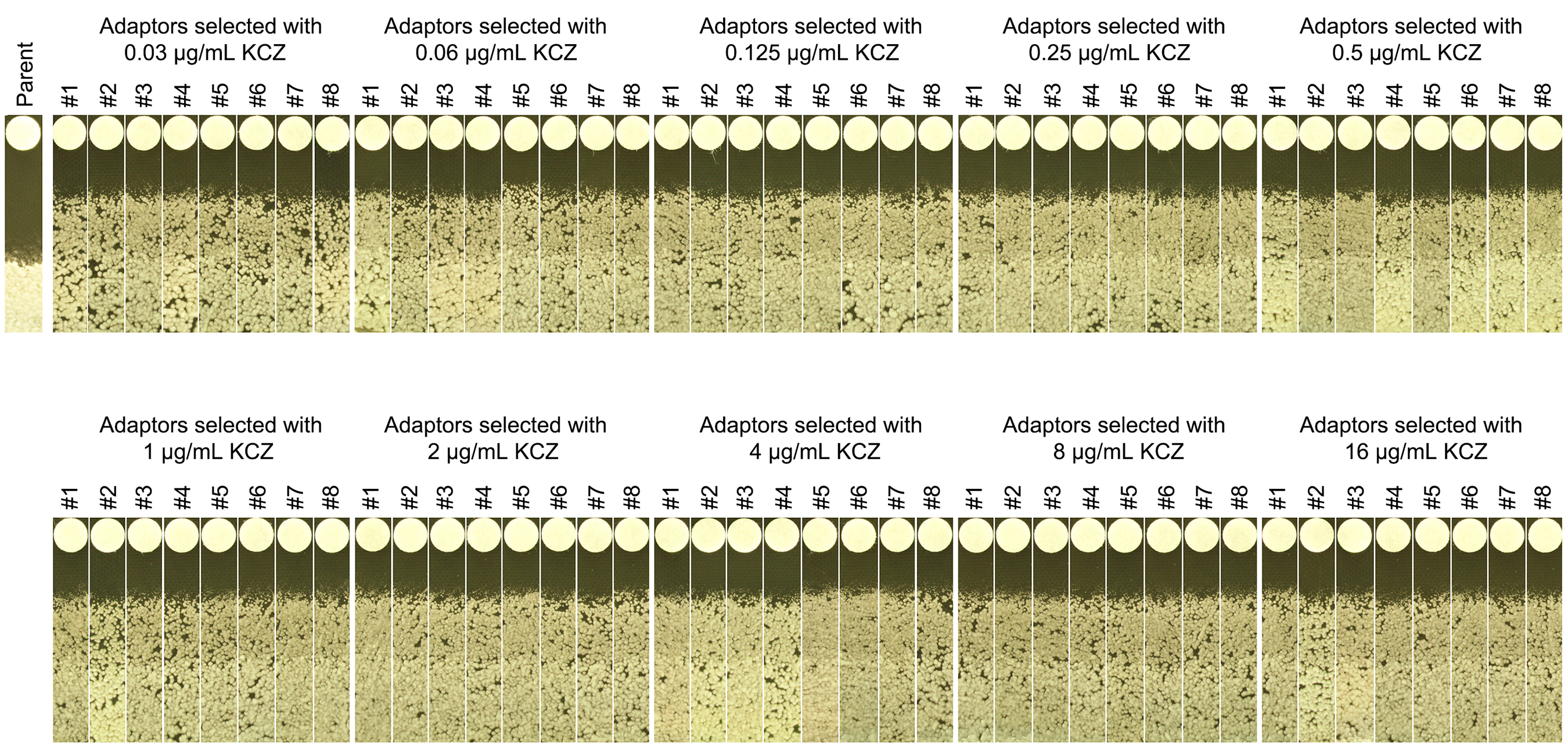

Supplement: Supplementary file 1 [file Data_Sheet_1.ZIP › Fig S1.tif]

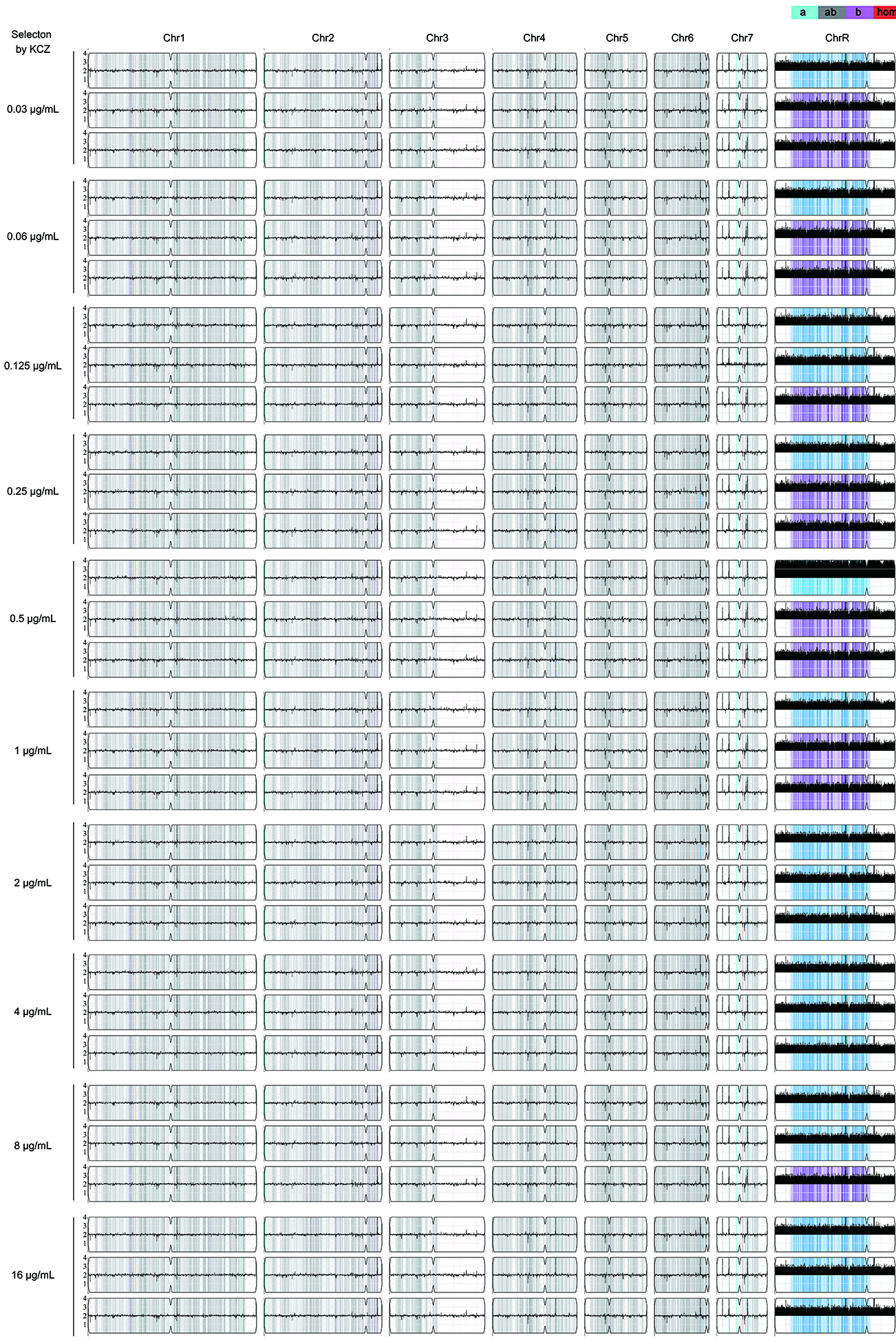

Supplement: Supplementary file 1 [file Data_Sheet_1.ZIP › Fig S2.tif]
